# Enhanced intrinsic functional connectivity in the visual system of visual artist: Implications for creativity

**DOI:** 10.3389/fnins.2023.1114771

**Published:** 2023-02-22

**Authors:** Tzu-Yi Hong, Ching-Ju Yang, Chung-Heng Shih, Sheng-Fen Fan, Tzu-Chen Yeh, Hsin-Yen Yu, Li-Fen Chen, Jen-Chuen Hsieh

**Affiliations:** ^1^Institute of Brain Science, College of Medicine, National Yang Ming Chiao Tung University, Taipei, Taiwan; ^2^Integrated Brain Research Unit, Division of Clinical Research, Department of Medical Research, Taipei Veterans General Hospital, Taipei, Taiwan; ^3^Department of Radiology, Taipei Veterans General Hospital, Taipei, Taiwan; ^4^Graduate Institute of Arts and Humanities Education, Taipei National University of the Arts, Taipei, Taiwan; ^5^Institute of Biomedical Informatics, College of Medicine, National Yang Ming Chiao Tung University, Taipei, Taiwan; ^6^Brain Research Center, National Yang Ming Chiao Tung University, Taipei, Taiwan; ^7^Department of Biological Science and Technology, College of Biological Science and Technology, National Yang Ming Chiao Tung University, Hsinchu, Taiwan; ^8^Center for Intelligent Drug Systems and Smart Bio-devices, National Yang Ming Chiao Tung University, Hsinchu, Taiwan

**Keywords:** visual artist, creativity, functional magnetic resonance imaging, resting state, functional connectivity, visual system

## Abstract

**Introduction:**

This study sought to elucidate the cognitive traits of visual artists (VAs) from the perspective of visual creativity and the visual system (i.e., the most fundamental neural correlate).

**Methods:**

We examined the local and long-distance intrinsic functional connectivity (FC) of the visual system to unravel changes in brain traits among VAs. Twenty-seven university students majoring in visual arts and 27 non-artist controls were enrolled.

**Results:**

VAs presented enhanced local FC in the right superior parietal lobule, right precuneus, left inferior temporal gyrus (ITG), left superior parietal lobule, left angular gyrus, and left middle occipital gyrus. VAs also presented enhanced FC with the ITG that targeted the visual area (occipital gyrus and cuneus), which appears to be associated with visual creativity.

**Discussion:**

The visual creativity of VAs was correlated with strength of intrinsic functional connectivity in the visual system. Learning-induced neuroplasticity as a trait change observed in VAs can be attributed to the macroscopic consolidation of consociated neural circuits that are engaged over long-term training in the visual arts and aesthetic experience. The consolidated network can be regarded as virtuoso-specific neural fingerprint.

## Introduction

Visual arts refer to a wide range of activities, including painting, sculpture, ceramics, design, crafts, photography, film, and architecture (Roodhouse, 2006). Many visual artists (VAs) develop an enhanced artistic capacity by training in the use of aesthetic elements (e.g., construction, composition, and abstraction) aimed at realizing their artistic and conceptual intentions (Lin et al., 2013). The aesthetic experience embodies the actions taken to appreciate aesthetic elements, as well as the emotions and bodily sensations that this elicits (Brinck, 2018). The ability of VAs to apply aesthetic experience and insight to the creation of artworks depends largely on their visual capacity (visual perception, visual memory, visual attention) and creativity (Chamberlain, 2017). Enhanced visual capability and visual creativity are perhaps the most prominent characteristics of VAs (Cupchik et al., 2009) and the visual system may be the neurological source of these abilities (Pepperell, 2011).

The visual attributes of any object are processed mainly in the occipital region of the brain (Chatterjee, 2003). Visual information is processed primarily along two pathways. The ventral pathway (what route: *vision for perception)* processes the identity of the object and tracks the features of the object, such as size, shape, and color (Zachariou et al., 2014). The ventral stream, leading from the posterior pole of the occipital cortex to the temporal lobe, is involved in identifying objects and tracking visual features. The dorsal stream, leading to the parietal lobe, is involved in spatial/motion analysis, object-directed actions, and visuomotor control (Goodale and Milner, 1992; Kravitz et al., 2013; Freud et al., 2016). The dorsal pathway (where route: *vision for action)* provides spatial awareness and the direction of movement (Schlegel et al., 2015). The dorsal pathway can be functionally subdivided into a dorsodorsal stream, which includes the superior parietal lobule (SPL, important for visuomotor control of actions) and a ventrodorsal stream, including the inferior parietal lobule (IPL), critical for the representation of complex actions (Peeters et al., 2013). Note however that a recent fiber tracking study disputes the existence of a specific dorsal pathway. They posited that in the processing of “where-information,” the angular gyrus (AG, BA39) channels the flow of information toward the middle temporal gyrus (MTG) in the visual cortex, and the inferior temporal gyrus (ITG) in the temporal cortex (Choi et al., 2020). AG is part of the default mode network involved in reading and comprehension, semantic processing, number processing, spatial cognition, memory retrieval, reasoning, and social cognition (Seghier, 2013). Converging multisensory information is combined and integrated in the AG to facilitate comprehension and give sense to events, manipulate mental representations, solve familiar problems, and reorient attention to relevant information (Seghier, 2013). Thus, the AG emerges as a cross-modal hub for the perception-to-recognition-to-action in visual art appreciation and creation (Seghier, 2013).

The process of creating artwork reflects VA’s creativity (Getzels and Csikszentmihalyi, 2020), thus creativity can be a core mental competence of VA. It appears that the process of creating visual artworks engages brain circuits that subserve the cognitive functions of attention, spatial arrangement, structural organization, motor planning, drawing skills, mnemonic storage, visuomotor processing, divergent thinking, mental imagery, self-consciousness, empathy, emotion regulation, face and object processing, and creativity (Locher, 2010). It also appears that the creative aspect of visual artwork production engages the temporal lobe, and particularly the inferior temporal cortex (e.g., fusiform gyrus) of the ventral pathway, which is involved in the formation of high-level complex visual information related to faces, places, objects, and scenes (Flaherty, 2005; Sugase-Miyamoto et al., 2011; Schaer et al., 2012; Conway, 2018; Beccone, 2020). It has been posited that artistic creativity is related object processing capacity along the ventral pathway (Kozhevnikov et al., 2013). It is plausible that long-term training in the visual arts strengthens the ventral pathway, manifesting as coherent activity within the neural networks associated with creativity (Miller et al., 1996; Petsche, 1996; Jung et al., 2010). The strength of intrinsic FC can be correlated with the visual creativity (Beaty et al., 2018).

Long-term professional training in the visual arts has been shown to enhance neurocognitive function and initiate changes in traits (resting state) in the brain. In a previous study using graph theory to assess functional connectivity (FC), we reported that the brain architecture of artists presents a hierarchical modular organization in which the brain states specific to specific artistic form mirror the mind states of virtuosos (Lin et al., 2013). In the current study, we examined intraregional (local) and interregional (long-distance) changes in FC, which are observable in resting-state brain oscillations (Wu et al., 2016). Regional homogeneity analysis (ReHo) (Zang et al., 2004) was used to estimate the local synchronization of brain activity as an indication of local connectivity, whereas the seed-based FC analysis (Yan et al., 2013) on the regions unveiled by ReHo was used to study long-range connectivity. It is commonly assumed that an increase in synchrony is indicative of local functional integration, whereas a decrease in synchrony is indicative of local functional segregation (Wu et al., 2016). An increase in long-range FC denotes functional integration between brain regions, whereas a decrease in long-range FC denotes functional segregation between brain regions (Fair et al., 2007). It has been reported that the ReHo approach provides superior seed localization, which is beneficial to seed-based FC analysis (Yan et al., 2013).

In the current study, we hypothesized that VAs should differ from controls in the neurodynamics of the visual system, and that the intrinsic strength of FC should be correlated with visual creativity (Beaty et al., 2018). Our results revealed that long-term training in the visual arts can consolidate the visual system at the macroscopic level, as evidenced by enhanced visuospatial capacity, visual attention, visuomotor control, and visual creativity. In other words, attuned neurodynamics is an indication of resilient plasticity nurtured through long-term experience.

## Materials and methods

### Participants

This study recruited 27 healthy university students majoring in the visual arts (VA, mean age 24.0 ± 1.7, 5 men) and 27 healthy non-artists matched for age and education (control group: CON mean age 23.2 ± 1.6, 4 men). The creative mediums of students in the VA group included oil paints, ink, sculpture materials, and/or multimedia. The average duration of artistic training was 11.07 ± 4.6 years. Students in the CON group had no more than 3 years of institutional training in the visual arts. All participants self-reported right-handedness without metal implants, brain damage, or neuropsychiatric diseases. The Beck Depression Inventory (BDI) (Beck et al., 1996) and the Beck Anxiety Inventory (BAI) (Beck and Steer, 1990) were used to exclude participants with obvious emotional liability. We also used the Wechsler Abbreviated Scale of Intelligence (WASI-III) (Chen and Chen, 2002) to ensure correspondence between the two studied groups in terms of general intelligence. This study was conducted in accordance with the Declaration of Helsinki and was approved by the Institutional Review Board of Taipei Veterans General Hospital with written informed consent obtained from all participants.

### Psychological measurements

This study was part of a project on neuroaesthetics, which addressed issues pertaining to domain-general and domain-specific neural organization among art students in a variety of fields (visual arts, dance, piano, strings, vocals, percussions) and non-artist healthy controls. To facilitate inter-group comparisons, we had all of the artists undergo the same psychological assessments and neuroimaging using the same scanning protocols. In the current study, we focused exclusively on VAs and controls. Creativity can be a core mental competence of VA since the process of creating artwork reflects VA’s creativity (Getzels and Csikszentmihalyi, 2020). Thus, all participants took the self-reported 40-item Chinese version of the Abbreviated Torrance Test for Adults (ATTA) to assess their aptitude in tasks involving visual (figural) and verbal manipulation (Chen, 2006). The ATTA is commonly used for cross-artist group comparisons (not addressed in the current study); however, it includes a figural part that engages creative drawing mirroring the visual art training of VAs. The ATTA measures the ability to think creatively in terms of fluency, originality, elaboration, and flexibility (Chen, 2006). Fluency refers to the number of ideas that a participant can generate in a limited time. Originality indicates one’s ability to create unique ideas. Elaboration indicates the ability to embellish ideas with details. Flexibility indicates one’s ability to generate many different ideas (Althuizen et al., 2010; Shen and Lai, 2014). The ATTA creativity index (CI) score refers to the sum of the four capacity scores. We followed standard protocols in administering and scoring the tests (Chen, 2006). SPSS Statistics (v. 23.0, IBM Corp., Armonk, NY, USA) was used for all psychological evaluation analyses. The results of the psychological evaluations were considered significant at *p* < 0.05.

### Data acquisition

Resting-state fMRI data were acquired using a 3T MAGNETOM Trio™ (Siemens, Erlangen, Germany) at the National Yang-Ming University. During scanning, participants lay supine with their heads fixed using foam cushions to minimize head motion. Scanning was performed in a T2*-weighted echo-planar imaging (EPI) sequence with the following parameters: 40 axial slices, TR = 2500 ms, TE = 30 ms, flip angle = 90°, FOV = 220 mm × 220 mm, slice thickness = 3.4 mm, matrix size = 64 × 64, and voxel size = 3.4 mm × 3.4 mm × 3.4 mm. A total of 200 contiguous functional volumes were collected from each participant. High-resolution T1-weighted 3D structural images were acquired using a magnetization-prepared rapid acquired gradient echo sequence [MPRAGE; repetition time (TR)/echo time (TE) = 2530 ms/3.03 ms, flip angle = 70°, field of view (FOV) = 224 mm × 256 mm × 192 mm, in-plane matrix size = 224 × 256 × 192, in-plane resolution = 1 mm]. All subjects were instructed to relax, remain still with their eyes open, think of nothing, and refrain from moving or falling asleep. All participants received brief training on how to focus their attention through breathing before scanning began, and all participants maintained a similar state throughout the actual experiment.

### Data analysis: Preprocessing

Data preprocessing was performed using the Data Processing Assistant for Resting-State fMRI (DPARSF) V4.5 Advanced Edition (State Key Laboratory of Cognitive Neuroscience and Learning, Beijing Normal University, China), which is based on the Data Processing and Analysis of Brain Imaging (DPABI) Toolbox version 4.1^1^ (Yan et al., 2016), with statistical parametrical mapping 12 (SPM 12; Wellcome Trust Center for Neuroimaging, University College London, London, UK) in Matlab 2015b (MathWorks, Inc., Natick, MA, USA). Based on experience in previous studies, the preprocessing of functional images was performed as follows: (1) slice timing correction; (2) realignment of images to the mean volume for correction of head motion; (3) co-registration to map functional information of resting fMRI images into an anatomical space (T1-weighted images) *via* intra-subject spatial alignment; and (4) segmentation of gray matter, white matter (WM) and cerebrospinal fluid (CSF) from coregistered T1 images using the unified segmentation model (Wu et al., 2016). Subjects with any instances of head movement exceeding 2 mm or 2° were excluded from further processing. The following nuisance variables were regressed: (1) six parameters of head movement calculated based on head motion with the Friston 24-parameter model translation and rotation during realignment in SPM12 (Friston et al., 1996); (2) the mean signal within the lateral ventricles for cerebral spinal fluid; and (3) the mean signal within a deep white matter region (centrum ovale). The images were normalized to the custom template from T1 weighted images of all subjects developed by the Montreal Neurological Institute (MNI) with resampled voxels at 2 mm × 2 mm × 2 mm. The resulting time series in each voxel was then linearly detrended and bandpass filtered (0.01–0.1 Hz) to extract low-frequency oscillations. Global signal regression (GSR) was not performed as it has been shown to exaggerate negative correlations (Murphy et al., 2009; Weissenbacher et al., 2009) and/or to distort group differences (Saad et al., 2012). We used WFU Pick Atlas toolbox^2^ to generate a visual system template based on the modified human visual pathway model by Choi et al. (2020). The visual system template includes the visual area [V1, V2, V3, V4, and V5/MT (BA 17, BA 18, and BA19)], inferior temporal area (BA 20), angular gyrus (BA 39), supramarginal gyrus (BA 40), and superior parietal lobule (BA 5, BA 7).

### Data analysis: ReHo analysis

ReHo maps were computed using Kendall’s coefficient of concordance (KCC) of the time series between a given voxel and its nearest neighbors (26 voxels) in a voxel-wise manner (Zang et al., 2004). The ReHo map of each subject was divided by its own global mean and then spatially smoothed using a 3D Gaussian kernel with 6 mm full width at half maximum (FWHM). Comparisons between groups of ReHo maps masked by the visual system template were examined using a two-sample *t*-test in SPM. The peaks of significant clusters were then selected as ReHo-based seeds. Statistical significance was set at an uncorrected voxel level of *p* < 0.005, followed by the family wise error (FWE)-corrected cluster level of *p* < 0.05.

### Data analysis: ReHo-seeded FC analysis

The preprocessing procedures were the same as those for the ReHo analysis, except for spatial smoothing, which was performed using a 6-mm FWHM Gaussian kernel prior to ReHo-seeded FC analysis. We observed significant between-group differences in terms of ReHo-seeded FC masked by the visual system template in all regions of interest (ROIs). Mean time-series activity was extracted within the spherical ReHo-seeded regions (5 mm radius) (Yan et al., 2013). ReHo-seeded FC was assessed between ROIs and the whole brain in a voxel-wise manner. Individual FC maps were then generated by computing the Pearson’s correlation coefficient (*r*) between the seeds and the related brain regions. After calculating the correlation between the reference time course and the time course of each voxel in the brain, the *r*-values were converted into *z*-values using Fisher’s r-to-z transformation to normalize the distribution. When analyzing differences between groups, two sample *t*-tests were performed on the ReHo-seeded FC maps of each seed with significance set at an uncorrected voxel level of *p* < 0.001, followed by an FWE corrected cluster level of *p* < 0.05 in SPM. Bonferroni corrections were made for multiple comparisons by adjusting the *p*-value divided by the number of seeds analyzed.

### Data analysis: Correlation analysis

VAs can be considered skilled experts in creative production (Degarrod, 2016); therefore, we used SPSS statistical software (v. 23.0, IBM Corp., Armonk, NY, USA) to compare the VA and CON groups in terms of the correlation between ReHo and ReHo-seeded FC maps (masked by the visual system template) and ATTA scores. Training effects among VAs were revealed by correlating variables in visual arts training (duration of visual arts training, duration of daily practice hours, and average amount of practice time per week) with ReHo and ReHo-seeded FC maps, respectively. We extracted the z-scores of significant peaks from individual ReHo and ReHo-seeded FC maps to perform group comparisons and assess two-tailed correlations between the z-scores and ATTA scores and variables of visual art training, respectively. The significant level was thresholded at *p* < 0.05.

## Results

### Demographic data and psychological evaluations

We observed no significant differences between the groups in terms of age, sex, level of education, depression, anxiety and intelligence. Compared to the control group, the VA group presented significantly higher scores for visual (figural) creativity (VA: 5.52 ± 2.06, CON: 3.03 ± 2.03, *p* < 0.001), fluency (VA: 16.1 ± 1.6, CON: 15.1 ± 1.7, *p* = 0.044), elaboration (VA: 17.5 ± 1.8, CON: 15.3 ± 2.4, *p* = 0.004), and flexibility (VA: 15.4 ± 1.6, CON: 14.5 ± 1.6, *p* = 0.041). They also presented a higher ATTA CI (sum of measurements of four categories of creative capacity; VA: 72.3 ± 6.1, CON: 65.0 ± 8.0, *p* < 0.001). Note that the VA group demonstrated also a sub-significant trend of higher originality performance as compared to the control group (VA: 17.0 ± 2.2, CON: 15.8 ± 2.6, *p* = 0.075) (Table 1).

**TABLE 1:** Demographic data and psychological results.

	VAs	CONs	*p*-value
	(*n* = 27)	(*n* = 27)	
Age (years)	24.0 ± 1.6	23.2 ± 1.6	0.88
Sex (male/female)	5/22	4/23	0.67
Duration of learning (years)	11.1 ± 4.6	–	–
Duration of daily practice (hours)	3.4 ± 2.1	–	–
Duration of weekly practice (hours)	20.5 ± 13.0	–	–
Education (years)	16.8 ± 1.6	16.3 ± 1.2	0.14
WAIS-III	110.9 ± 7.3	109.51 ± 7.22	0.55
BDI	8.6 ± 7.3	7.3 ± 6.8	0.42
BAI	8.5 ± 5.7	5.9 ± 4.7	0.054
ATTA Creativity index	72.3 ± 6.1	65.0 ± 8.0	<0.001***
Verbal creativity	1.20 ± 0.9	0.78 ± 0.71	0.54
Visual creativity	5.52 ± 2.06	3.03 ± 2.03	<0.001***
Fluency	16.1 ± 1.63	15.1 ± 1.66	0.044*
Originality	17.0 ± 2.2	15.8 ± 2.6	0.075
Elaboration	17.5 ± 1.8	15.8 ± 2.4	0.004**
Flexibility	15.4 ± 1.6	14.5 ± 1.6	0.041*

Data expressed as mean ± standard deviation.

*p < 0.05, **p < 0.01, ***p < 0.001.

VA, visual artist; CON, control; WAIS-III: Wechsler Adult Intelligence Scale-III; BDI, Beck Depression Inventory; BAI, Beck Anxiety Inventory; ATTA, Abbreviated Torrance Test for Adults.

### Altered local connectivity in the visual system of VAs

**FIGURE 1:**
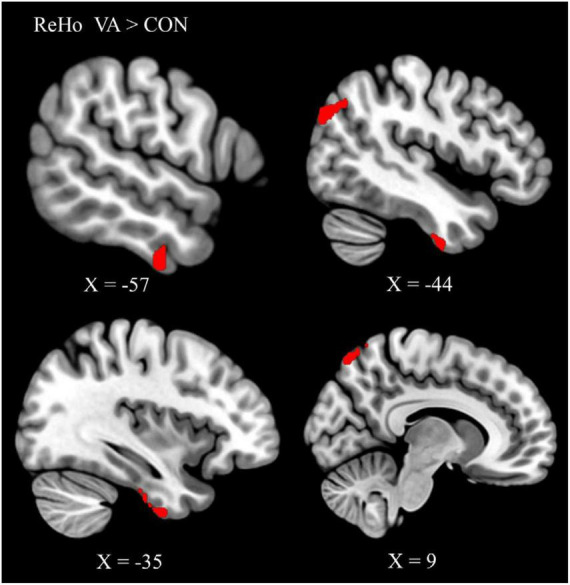
Between-group differences in ReHo: ReHo in the VA group (red) was higher than in the CON group in three regions: Rt. SPL, Rt. precuneus, Lt. ITG/FG, Lt. AG, and Lt. MOG. ReHo, regional homogeneity; VA, visual artist; CON, control; Lt., left; Rt., right; SPL, superior parietal lobule; ITG, inferior temporal gyrus; FG, fusiform gyrus; AG, angular gyrus; MOG, middle occipital gyrus.

To identify the cardinal functional hubs of the VAs, we quantified intraregional functional integration/segregation by calculating the voxel-wise ReHo value. The ReHo value was significantly higher in the VA group than in the CON group in the right SPL, right precuneus, left ITG, left ITG/fusiform gyrus (FG), left AG, and left middle occipital gyrus (MOG) of the visual system (Figure 1 and Table 2). These regions are associated with visual imagery (Ishai et al., 2000; Mechelli et al., 2004), visuospatial processing (Tres and Brucki, 2014), and the perception of objects, faces, and scenes (Sugase-Miyamoto et al., 2011; Conway, 2018). Notably, these regions are also engaged in elementary constructs of visual productivity (e.g., visual capacity, formation of higher-level complex visual representation, divergent thinking, long-term memory storage, and visual imagery) (Miyashita, 1993; Ishai et al., 2000; Mechelli et al., 2004; Sugase-Miyamoto et al., 2011; Zhang et al., 2011).

**TABLE 2:** Regions showing differences in ReHo between VAs and CONs.

Brain region	BA	MNI coordinate			Size	*t* score
		*x*	*y*	*z*		
**VAs > CONs**						
Right SPL*	7	9	–72	60	203	4.10
Right Precuneus*	7	3	–60	66		3.85
Left ITG*	20	–57	–12	–36	150	4.32
Left ITG/FG*	20	–35	–18	–33		3.85
Left AG	39	–50	–72	36	115	3.22
Left MOG	19	–42	–80	30		3.06

Size refers to the number of voxels in the cluster (peak level uncorrected *p* < 0.005, cluster level corrected FWE, *p* < 0.05). ReHo, regional homogeneity; VA, visual artist; CON, control; BA, Brodmann area; SPL, superior parietal lobule; ITG, inferior temporal gyrus; FG, fusiform gyrus; AG, angular gyrus; MOG, middle occipital gyrus.

*Also peak level uncorrected *p* < 0.001, cluster level corrected FWE, *p* < 0.05.

### Altered long-range functional connectivity in the visual system of VA

**FIGURE 2:**
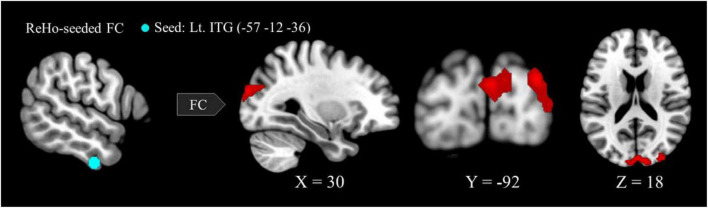
Between-group differences in ReHo-seeded FC: The VA group presented stronger FC in the Lt. ITG (light blue)-bilateral cuneus (red), -bilateral MOG, and -bilateral SOG. ReHo, regional homogeneity; FC, functional connectivity; VA, visual artist; Lt., left; Rt., right; ITG, inferior temporal gyrus; MOG, middle occipital gyrus; SOG, superior occipital gyrus.

Six regions of interest (ROIs) identified in ReHo analysis were used as seeds for FC analysis. The VA group presented a higher FC of the left ITG-right cuneus, -right MOG, and -bilateral SOG (Figure 2 and Table 3). These targeted regions are associated with the functional processing of visual characteristics of objects and scenes, object recognition, and form representation.

**TABLE 3:** Differences in ReHo-seeded FCs between VAs and CONs.

Brain region	BA	MNI coordinate			Size	*t* score
		*x*	*y*	*z*		
**VAs > CONs [Seed: Left ITG (–57 –12 –36)]**						
Right cuneus	18	3	–93	18	138	4.55
Right SOG	18	21	–87	6		4.36
Left SOG	17	–9	–99	12		4.35
Right MOG	19	30	–93	16	107	4.54
Right MOG	19	43	–81	6		3.98

Size refers to the number of voxels in the cluster (peak level uncorrected *p* < 0.001, cluster level corrected FWE, *p* < 0.05). Bonferroni corrections were made for multiple comparisons by adjusting the 0.05 divided by 6 of the seeds analyzed. ReHo, regional homogeneity; FC, functional connectivity; VA, visual artist; CON, control; BA, Brodmann area; ITG, inferior temporal gyrus; MOG, middle occipital gyrus; SOG, superior occipital gyrus.

### Between-group differences in the correlation between FC strength and creativity

**FIGURE 3:**
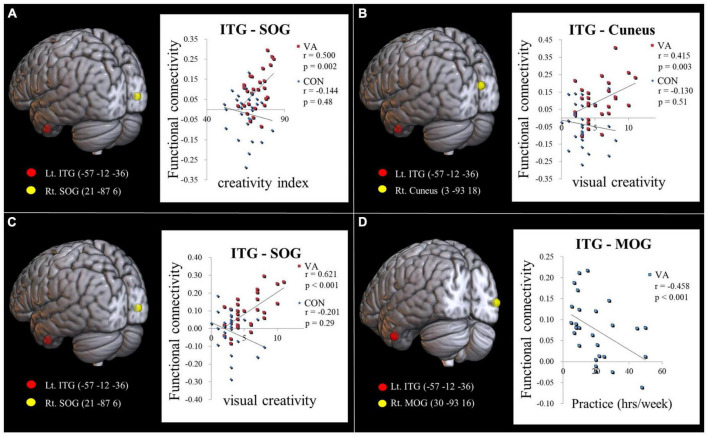
Examining the link between functional connectivity strength, ATTA scores, and practice time. **(A)** The strength of the Lt. ITG-Rt. MOG FC is positively correlated with the creativity index measured by the ATTA score among VAs. **(B)** The strength of the Lt. ITG -Rt. Cuneus FC is positively correlated with the visual creativity score of the ATTA among VAs. **(C)** The strength of the Lt. ITG -Rt. SOG FC is positively correlated with the visual creativity score of the ATTA among VAs. There is no significant correlation observed among CONs in **(A–C)**. **(D)** The strength of the Lt. ITG -Rt. MOG FC is negatively correlated with weekly practice time among VAs. The significance level is thresholded at *p* = 0.05. ATTA, Abbreviated Torrance Test for Adults; Lt., left; Rt., right; VA, visual artist; CON, control; ITG, inferior temporal gyrus; MOG, middle occipital gyrus; SOG, superior occipital gyrus; FC, functional connectivity.

Our hypothesis posited that the VA group would display distinct neurodynamics in the visual system compared to the CON group. We also expected the strength of FC to be associated with visual creativity score, based on prior research (Beaty et al., 2018). In our study, we found a significant positive correlation (*r* = 0.500, *p* = 0.002; Figure 3A) between the CI score and the strength of FC between the left ITG and the right SOG in the VA group. However, no significant correlation was observed in the CON group (*r* = −0.144, *p* = 0.48; Figure 3A). Furthermore, within the VA group, we discovered a significant positive correlation between the visual creativity score and the strength of FC between the left ITG and the right cuneus (*r* = 0.415, *p* = 0.003; Figure 3B), as well as the strength of FC between the left ITG and the right SOG (*r* = 0.621, *p* < 0.001; Figure 3C). However, no significant correlations were observed in the CON group between the visual creativity score and the strength of FC between the left ITG and the right cuneus (*r* = −0.130, *p* = 0.51; Figure 3B) and the strength of FC between the left ITG and the right SOG (*r* = −0.201, *p* = 0.29; Figure 3C). Moreover, we noted a significant negative correlation (*r* = −0.458, *p* < 0.001) between the weekly practice duration (measured in hours) and the strength of FC between the left ITG and the right MOG in the VA group (Figure 3D). There were no notable differences between the VA and CON groups concerning the correlation between the strength of intraregional FC and behavioral variables (training duration, daily practice hours, and psychological measurements). Additionally, in the VA group, no significant correlations were observed between the strength of interregional FC and other behavioral variables (*p* > 0.05).

We observed no between-group differences in terms of the correlation between intraregional FC and other psychological measurements. In the VA group, we observed no correlations between interregional FC and the duration of training, daily practice hours, or other ATTA subscales (*P* > 0.05).

## Discussion

Training in the visual art and the cultivation of aesthetic sensibilities can shape the brain of VAs. In the current study, we sought not to activate any cognitive processes related to the active creation of visual artwork. Instead, we posited that in the context of functional connectivity, the consolidation of neural circuits that engage during long-term learning may underpin the macroscopic neuroplasticity of VAs. This study reports a neurosignature representative of the neural makeup in the visual system of VAs (e.g., visual perception, visual attention, and visual creativity).

### Constellations of local and long-range connectivity in the visual system relate to the capacity of VAs in visual arts production and appreciation

The IPL (particularly the AG) has been proposed as a major “store house” of artistic creativity (Chakravarty, 2012). Creative cognition and creative output as conceived in the prefrontal cortex (ventromedial and dorsolateral prefrontal cortex, respectively) are relayed to the IPL and SPL reciprocally with the perceptive visual system (dorsal and ventral pathways) for both artistic production and appreciation. The ventromedial prefrontal cortex is involved in aesthetic evaluation and appreciation (Chakravarty, 2012). The VA group presented superior intraregional functional integration in terms of higher ReHo value (Wang et al., 2011) in the neural substrates of both the dorsal pathway (right SPL, right precuneus, left AG) and the ventral pathway (left ITG/FG) (Jiang and Zuo, 2016; Table 2 and Figure 1). The SPL and AG of the dorsal pathway (involved in artistic spatiality) (Seydell-Greenwald et al., 2017) serves spatial awareness, attention, spatial/motion analysis, goal-directed action, and visuomotor control (Husain and Nachev, 2007; Shomstein, 2012; Seghier, 2013). The precuneus and SPL also participate in visual imagery and other visuospatial processing (Cavanna and Trimble, 2006; Walker et al., 2011; Tres and Brucki, 2014).

As a key region connecting the occipital and parietal cortices, the ITG plays critical roles in the perception of objects, faces, and scenes (Conway, 2018) as well as in visual creativity (e.g., visual capacity, formation of higher-level complex visual representation, divergent thinking, long-term memory storage, and visual imagery) (Miyashita, 1993; Ishai et al., 2000; Mechelli et al., 2004; Sugase-Miyamoto et al., 2011; Zhang et al., 2011). The pattern of higher intrinsic FC of the left ITG-right cuneus, -right MOG, and -bilateral SOG (Table 3 and Figure 2) in the VA group is substantiated by the anatomical and structural connectivity studies of the visual system (Zhang et al., 2013; Takemura et al., 2017; Lin et al., 2020). We considered that the constellations of local and long-range connectivity changes implicate functional synergy between brain regions and can be better appreciated in the context of the neurological underpinning of visual artistic production and appreciation (Smith et al., 2003; Vartanian and Goel, 2004; Kozbelt and Seeley, 2007; Kravitz et al., 2013; Vessel et al., 2019).

### FC strength in the visual system of VAs mirrors creativity

VA group was of higher creativity as compared to the control group could be evidenced by their higher ATTA scores. In the VA group, we observed a significantly positive correlation between the ATTA CI score and the strength of the left ITG-right SOG FC (Figure 3A). When we consider that the ATTA CI score is a sum of four creative capacities (fluency, originality, elaboration, and flexibility), these findings indicate the consolidation of the ventral pathway in VAs facilitates the integration of various abilities in the creative process. These findings echo those in a previous study in which it was reported that network attributes in the occipital regions are predictive of individual differences in creative ability (Jiao et al., 2017).

The positive correlations between FC strength in the left ITG (with respective right cuneus and right SOG) and visual creativity score in the VA group (Figures 3B, C) partly indicate the neural underpinnings of creativity in VAs. Visual creativity refers to the appreciation and ability to produce novel esthetically pleasing visual forms (e.g., sketches, paintings, and graphic design). The process of creating these forms depends heavily on visual imagery (Heilman et al., 2003; Sack et al., 2008; Pelowski et al., 2017) and the ability to combine disparate visual representations to form new entities. The production and appreciation of visual products are subserved by the two visual pathways (Chakravarty, 2012). These results confirm that the creation of artworks depends on the visual system and particularly the ITG of the ventral pathway, which controls visual imagery, visual perception (Ishai et al., 2000), and visual attention for object recognition (Zhang et al., 2011). Object visualization ability (the ability to construct visual appearances of objects in terms of their shapes, color, and texture) in the ventral pathway and spatial visualization ability (the ability to spatial awareness and direction of movements) in the dorsal pathway both contribute to artistic creativity (Kozhevnikov et al., 2010, 2013). Scores on ATTA emphasize object visualization ability over spatial visualization ability to enrich the creative content. Our data indicates that the ventral pathway is particularly consolidated in VAs, due to strengthening of the FC between the ITG and the cuneus/SOG following long-term practice in bringing artistic notions to fruition.

### Learning effect was negatively correlated with connectivity strength

Extensive training can lead to “effortless doing,” which manifests neurologically as diminished brain activity, neural oscillation, and functional connectivity (Sampaio-Baptista et al., 2015; Ji et al., 2017). One previous study of elite athletes discovered that lower amplitude signals (low- and high-frequency alpha event related desynchronization) in the ventral pathway (in the occipital and temporal areas) are an indication of processing that is more efficient than that observed in normal individuals (Babiloni et al., 2009). High-efficiency processing is characterized by a bidirectional reduction of activation in areas associated with task execution and the deactivation of regions associated with the processing of irrelevant information (Qiu et al., 2019). It has been suggested that the ITG is involved in visual object recognition and visual perception, whereas the MOG is primarily involved in the characterization of objects in terms of shape and category (Proklova et al., 2016). The occipital and temporal regions together co-constitute the ventral visual pathway (“what” route) for object recognition (Kravitz et al., 2013). The negative correlation between FC strength in the left ITG-right MOG and the duration of weekly practice (as an indicator of practice intensity) (Figure 3D) connotes “effortless doing” as a manifestation of proficiency in the execution of visual skills following the sustained practice of skills involving visual manipulation (Sheth and Young, 2016; Conway, 2018). Long-term training and the resulting proficiency in the low-level processes typically engaged in the visual arts provides a solid foundation for the creative production of artworks, which requires efficient cognitive engagement.

### Limitations and future directions

The current study has several *limitations*, which should be considered in the interpretation of our findings. First, only the ATTA was used as an indicator of general creativity. Future work should include other more specific creativity tasks (e.g., spatial visualization ability and creativity) to further explore the psychological manifestations of long-term training in the visual arts. Second, ReHo and ReHo seed-based FC can be used together to detect the synchronization of brain activity (local and long-distance) (Yan et al., 2013). The combination of these two approaches can help to identify cardinal hubs and facilitate seed selection for FC analysis (Zang et al., 2004). In the current study, we focused only on the visual system, as it is the most fundamental neural system for VAs. In the future, researchers could conduct a more thorough analysis of the FC and the neurodynamics of otherwise identified neural substrates and neural networks potentially involved in visual creativity (e.g., default mode network) (Beaty et al., 2014). These networks could be employed as heuristics by which to elucidate changes in the brain traits of VAs (Takeuchi et al., 2011).

## Conclusion

The resilience of the brain is largely due to the dynamic reconfiguration of functional organization to support a variety of cognitive demands. Learning-induced neuroplasticity as a trait change observed in VAs can be attributed to the macroscopic consolidation of consociated neural circuits that are engaged over long-term training in the visual arts and aesthetic experience. It appears that the visual creativity of VAs is correlated with the strength of intrinsic functional connectivity in the visual system. The consolidated network can be regarded as a virtuoso-specific neural fingerprint.

## Data availability statement

The original contributions presented in this study are included in the article/supplementary material, further inquiries can be directed to the corresponding authors.

## Ethics statement

The studies involving human participants were reviewed and approved by the Institutional Review Board of Taipei Veterans General Hospital. The patients/participants provided their written informed consent to participate in this study.

## Author contributions

T-YH: conceptualization, investigation, formal analysis, data curation, writing—original draft, and visualization. C-JY and C-HS: investigation. S-FF and H-YY: resources. T-CY: supervision. L-FC: conceptualization and supervision. J-CH: conceptualization, methodology, resources, writing—review and editing, supervision, project administration, and funding acquisition. All authors contributed to the article and approved the submitted version.

## Footnotes

1
http://rfmri.org/dpabi

2
https://www.nitrc.org/projects/wfu_pickatlas
