# Corrigendum: Enhanced intrinsic functional connectivity in the visual system of visual artist: implications for creativity

**DOI:** 10.3389/fnins.2023.1258987

**Published:** 2023-08-24

**Authors:** Tzu-Yi Hong, Ching-Ju Yang, Chung-Heng Shih, Sheng-Fen Fan, Tzu-Chen Yeh, Hsin-Yen Yu, Li-Fen Chen, Jen-Chuen Hsieh

**Affiliations:** ^1^Institute of Brain Science, College of Medicine, National Yang Ming Chiao Tung University, Taipei, Taiwan; ^2^Integrated Brain Research Unit, Division of Clinical Research, Department of Medical Research, Taipei Veterans General Hospital, Taipei, Taiwan; ^3^Department of Radiology, Taipei Veterans General Hospital, Taipei, Taiwan; ^4^Graduate Institute of Arts and Humanities Education, Taipei National University of the Arts, Taipei, Taiwan; ^5^Institute of Biomedical Informatics, College of Medicine, National Yang Ming Chiao Tung University, Taipei, Taiwan; ^6^Brain Research Center, National Yang Ming Chiao Tung University, Taipei, Taiwan; ^7^Department of Biological Science and Technology, College of Biological Science and Technology, National Yang Ming Chiao Tung University, Hsinchu, Taiwan; ^8^Center for Intelligent Drug Systems and Smart Bio-devices, National Yang Ming Chiao Tung University, Hsinchu, Taiwan

**Keywords:** visual artist, creativity, functional magnetic resonance imaging, resting state, functional connectivity, visual system

In the published article, there was an error in the legend for **Figure 3** as published. The **Figure 3** legend erroneously states “negatively correlated among CONs.” It should read “There is no significant correlation observed among CONs in (A–C).” The corrected legend appears below.

In the published article, there was an error in **Figure 3** as published. We had missed to insert a label in the published Figure 3. We have labeled the statistical *p*-values in the corrected paper to better the result display in **Figure 3**. The corrected Figure 3 appears below.

**Figure 3:**
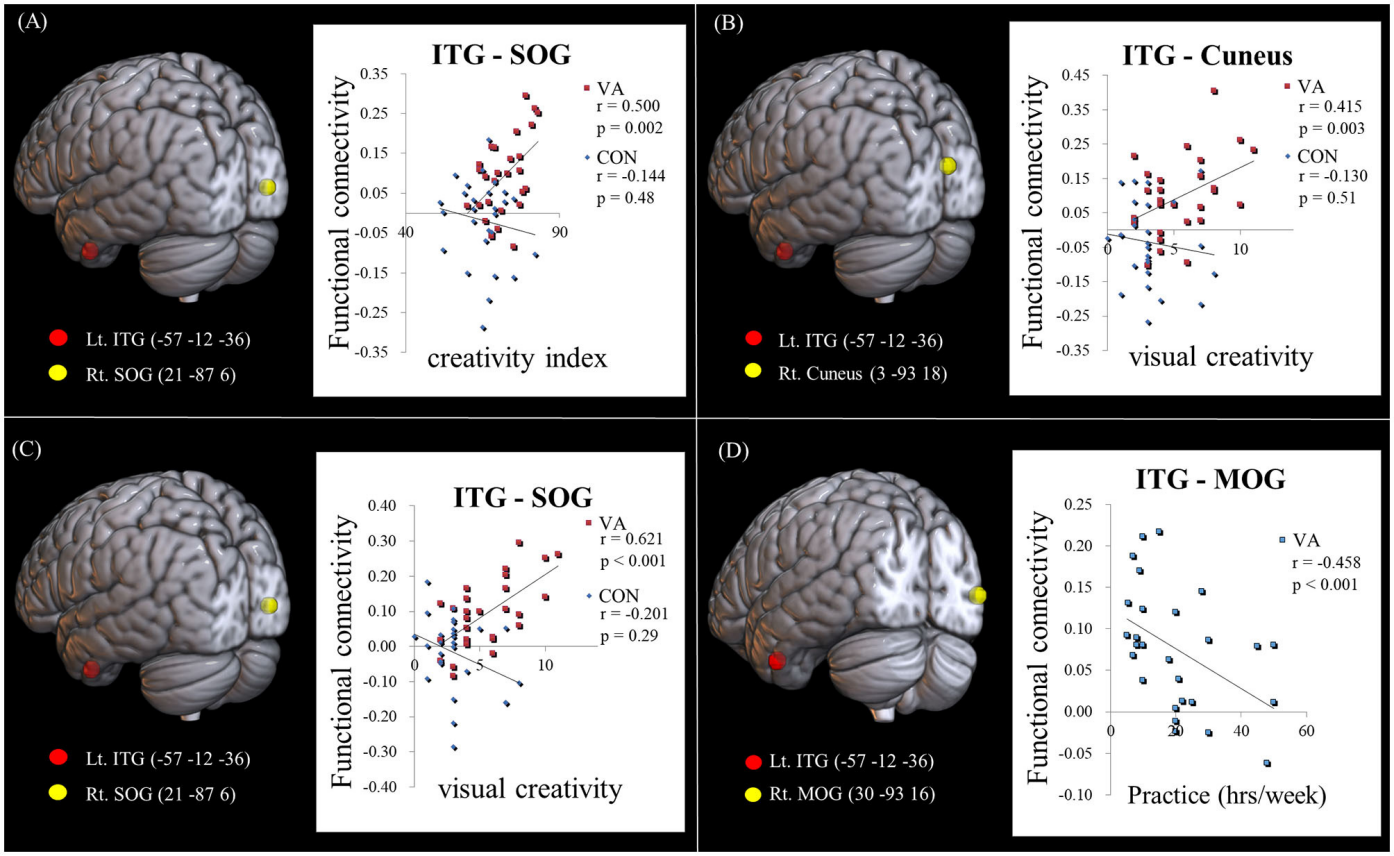
Examining the link between functional connectivity strength, ATTA scores, and practice time. **(A)** The strength of the Lt. ITG-Rt. MOG FC is positively correlated with the creativity index measured by the ATTA score among VAs. **(B)** The strength of the Lt. ITG -Rt. Cuneus FC is positively correlated with the visual creativity score of the ATTA among VAs. **(C)** The strength of the Lt. ITG -Rt. SOG FC is positively correlated with the visual creativity score of the ATTA among VAs. There is no significant correlation observed among CONs in **(A–C)**. **(D)** The strength of the Lt. ITG -Rt. MOG FC is negatively correlated with weekly practice time among VAs. The significance level is thresholded at *p* = 0.05. ATTA, Abbreviated Torrance Test for Adults; Lt., left; Rt., right; VA, visual artist; CON, control; ITG, inferior temporal gyrus; MOG, middle occipital gyrus; SOG, superior occipital gyrus; FC, functional connectivity.”

In the published article, there was an error in the Funding statement. We had missed to add one funding resource. We have made the amendment in the corrected article. The correct Funding statement appears below.
